# Cerebellar, brainstem and spinal cord metastases from esophageal cancer following radiotherapy: A case report and literature review

**DOI:** 10.3892/ol.2014.2130

**Published:** 2014-05-09

**Authors:** PENG ZHANG, WEI FENG, XIAO ZHENG, YUE-ZHEN WANG, GUO-PING SHAN

**Affiliations:** Department of Radiation Oncology, Zhejiang Key Laboratory of Diagnosis and Treatment Technology on Thoracic Oncology (Lung and Esophagus), Zhejiang Cancer Hospital, Hangzhou, Zhejiang 310022, P.R. China

**Keywords:** esophageal cancer, metastasis, cerebellum, spinal cord, brainstem

## Abstract

Cerebellar, brainstem and spinal cord metastases from esophageal cancer following radiotherapy are extremely rare. The current study presents the case of a 74-year-old male who was admitted to the Zhejiang Cancer Hospital (Hangzhou, China) with a poorly-differentiated neuroendocrine carcinoma of the esophagus. Following radiotherapy, multiple abnormal signals in the brainstem and spinal cord were found on magnetic resonance imaging (MRI). Following palliative radiochemotherapy, the clinical symptoms and abnormal MRI signals in the brainstem and spinal cord were found to improve. This case revealed that brain metastasis from esophageal carcinoma may occur simultaneously with brainstem and spinal cord metastases.

## Introduction

The overall rate of brain metastasis from gastrointestinal malignant tumors, including colorectal, gastric, pancreatic, esophageal and gastrointestinal cancer, is reported as 3–8% (1–3). Notably, esophageal carcinomas account for 1–5% of these brain metastases (4,5). Neuroendocrine carcinoma of the esophagus is a rare malignant tumor originating from the neuroendocrine cells. Depending on the degree of differentiation, the tumors are classified as carcinoid (well-differentiated), atypical carcinoid (moderately-differentiated) and neuroendocrine small cell carcinoma (poorly-differentiated). Neuroendocrine carcinomas account for 0.15–2.8% of esophageal carcinomas (6,7). According to previous studies, the pathological types of brain metastases originating from esophageal carcinoma are generally squamous carcinoma in Asian countries and adenocarcinoma in Western countries (4,5,8). The incidence of esophageal cancer in China is high, and reports of brain metastasis of esophageal cancer in China in the international literature are rare. Due to the low incidence and lack of specific clinical manifestations, brain metastases from esophageal carcinoma usually remain undiagnosed and are treated incorrectly. Cases of simultaneous metastases occurring in the cerebellum, brainstem and spinal cord are extremely rare. The current study reports such a case. Patient provided written informed consent.

## Case report

### Primary tumor (esophageal cancer) diagnosis and treatment

**Figure 1:**
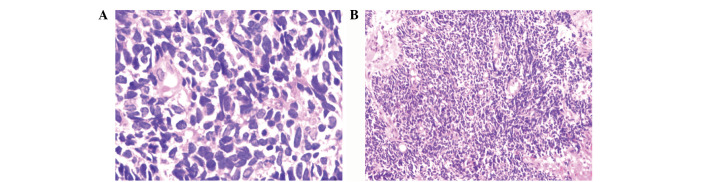
Pathological appearance of the primary esophageal tumor cells exhibiting little cytoplasm and heavily stained nuclei. Magnifications of (A) ×400 and (B) ×100 (hematoxyling and eosin stain).

**Figure 2:**
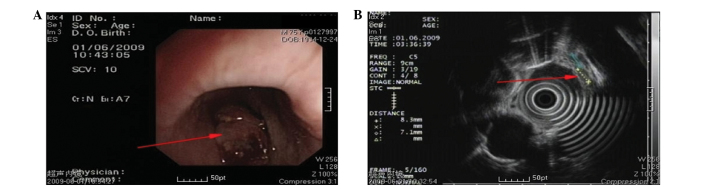
Endoscopic ultrasonography revealing (A) an esophageal neoplasm (indicated by the red arrow) and (B) multiple enlarged lymph nodes on the outside of the esophagus wall (indicated by the red arrow).

**Figure 3:**
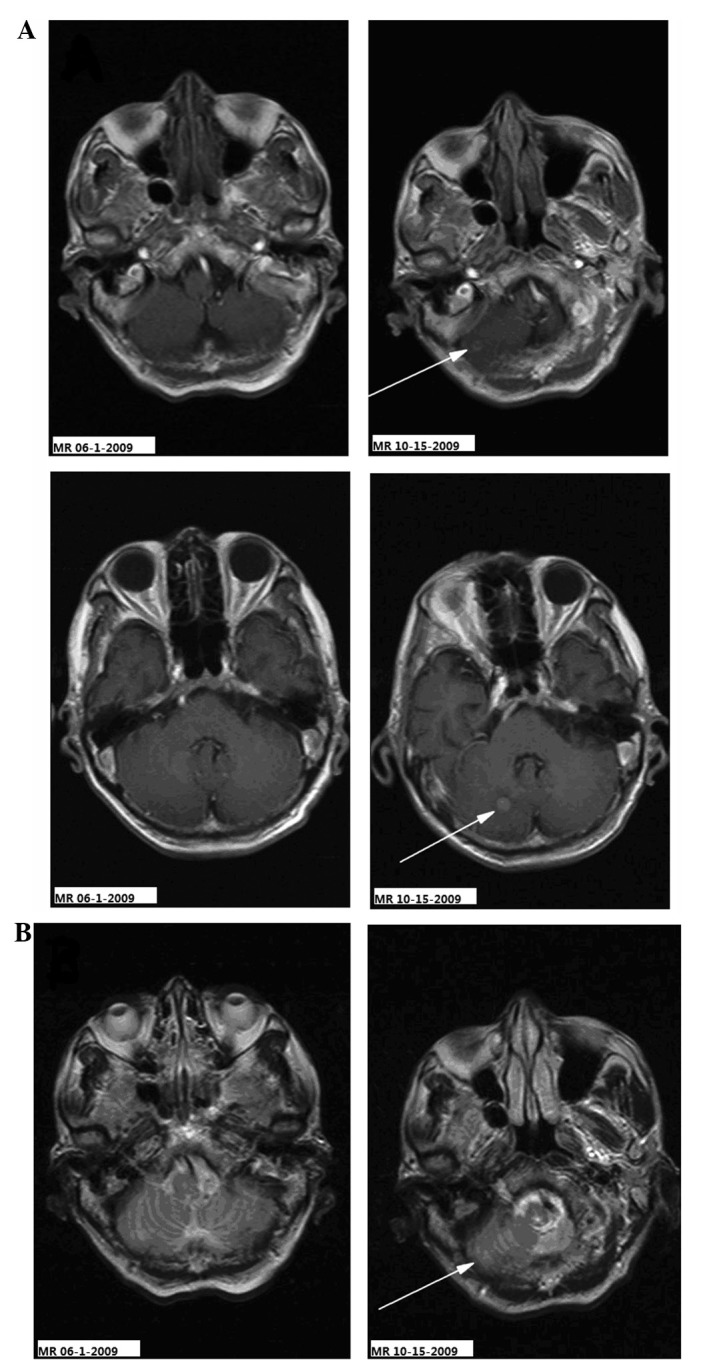
Comparison between the cerebral MR images of the esophageal cancer patient obtained prior to (MR 06-1-2009) and following (MR 10-15-2009) the metastasis. (A) Enhanced axial T1-weighted images and (B) axial T2-weighted images. Left (MR 06-1-2009), no abnormal signals were observed in the brain; and right (MR 10-15-2009), abnormal signals were observed in the right cerebellar hemisphere (indicated by the arrows). MR, magnetic resonance.

**Figure 4:**
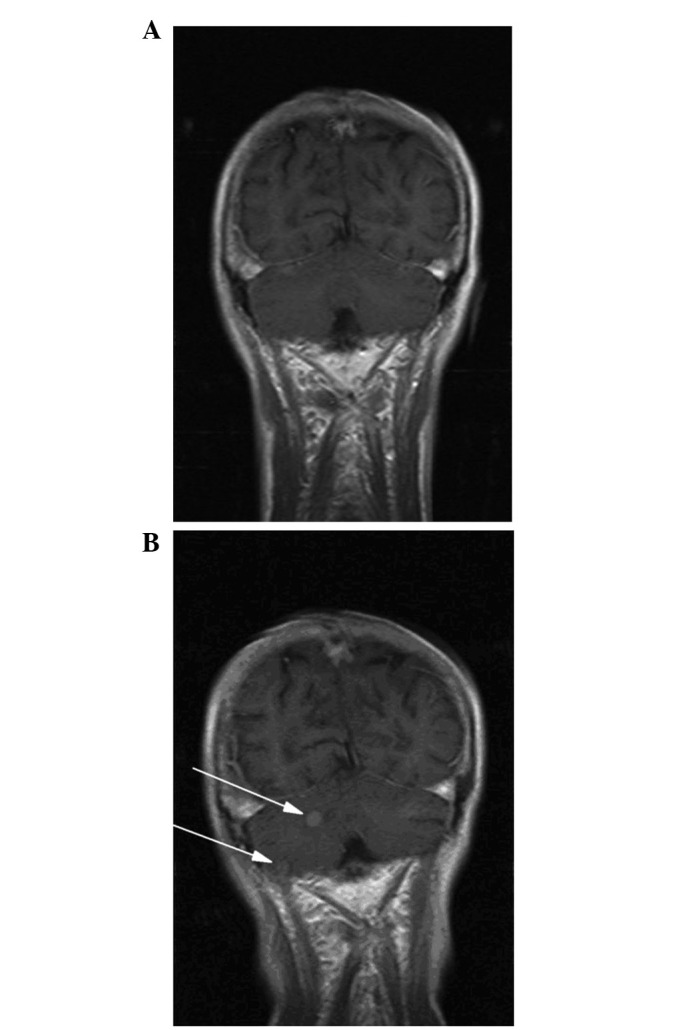
Comparison between the cerebral MR images obtained prior to and following the metastasis. Coronal T1-enhanced image showed (A) no abnormal signals in the brain (MR 06-1-2009) and (B) abnormal signals in the right cerebellar hemisphere (indicated by the two arrows; MR 10-15-2009). MR, magnetic resonance.

**Figure 5:**
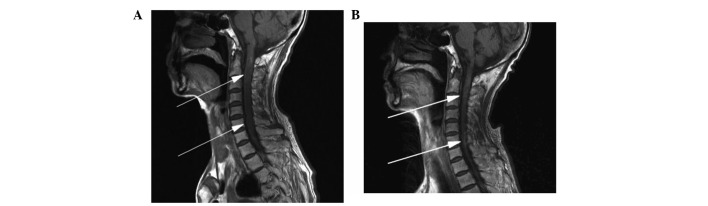
Comparison between the MR images of the cervical spinal cord prior to and following treatment. Sagittal T1-weighted images performed (A) prior to chemotherapy (MR 10-28-2009) showing abnormal signals in the medulla oblongata and cervical cord, considered to be the metastatic lesions (indicated by the two arrows), and (B) following chemotherapy (MR-11-23-2009) showing that the abnormal signals of the medulla oblongata and cervical cord had markedly improved (indicated by the two arrows). MR, magnetic resonance.

A 74-year-old male presented to the Zhejiang Cancer Hospital (Hangzhou, China) with complaints of swallowing difficulties that had been apparent for three months. Gastroscopy revealed the presence of two nodules of 1.5 cm in diameter, with deep ulceration and eruption of the surrounding esophageal mucous membrane, at 22–25 and 27–35 cm from the incisor teeth. Pathological evaluation indicated malignant epithelial tumors described as poorly-differentiated neuroendocrine carcinoma or basal cell squamous carcinoma (Fig. 1). Endoscopic ultrasound revealed damage to five layers of the esophageal wall, with lesions invading the outer membrane and muscular layer; multiple enlarged lymph nodes were also observed (Fig. 2). Ultrasound revealed supraclavicular lymph node metastasis. No abnormal signals were observed by contrast-enhanced magnetic resonance imaging (MRI) of the brain and via whole body bone imaging (Figs. 3–5). The diagnosis was of esophageal carcinoma with supraclavicular metastasis. Radiotherapy was administered to the esophageal lesions and supraclavicular area, with a total dose of 64 Gy/32 fractions of 2 Gy per fraction. The swallowing difficulty symptom improved, and a follow-up enhanced chest computed tomography (CT) scan revealed slight thickening of the upper segment of the esophagus following radiotherapy.

### Diagnosis of central nervous system metastasis and treatment

**Figure 6:**
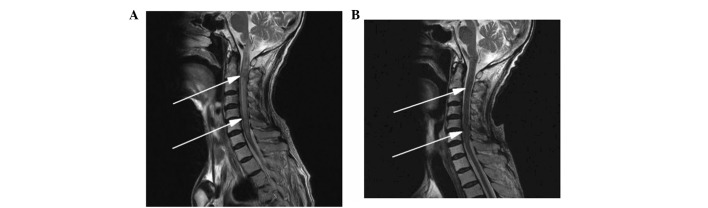
Comparison between the MR images of the cervical spinal cord prior to and following chemotherapy. Sagittal T2WI (A) prior to chemotherapy (MR 10-28-2009) showing increased T2WI signals and abnormal morphological augmentation in the medulla oblongata and cervical cord indicating metastatic lesions (indicated by the two arrows), and (B) following chemotherapy (MR 11-23-2009) showing decreased T2WI signals in the medulla oblongata and cervical cord consistent with the signals of normal tissues, and marked improvement in the abnormal morphology (as indicated by the two arrows). MR, magnetic resonance; T2WI, T2-weighted image.

**Figure 7:**
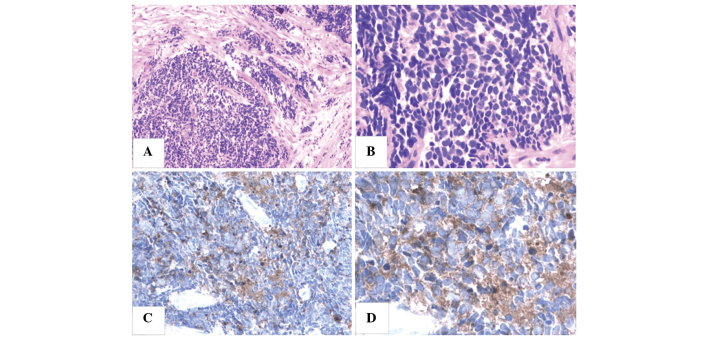
(A) Tumor cells characteristically concentrated into canalicular and branching structures (H&E stain; magnification, ×100). (B) Tumor cells exhibiting little cytoplasm, heavily stained nuclei and lacking evident nucleoli (H&E stain; magnification, ×400). Tumor cells showing cytoplasmic positive staining for synaptophysin using the diaminobenzidine method at magnifications of (C) ×200 and (D) ×400. H&E, hematoxylin and eosin.

The patient complained of constant occipital headache with slight pain in the bilateral upper arm and fatigue, but no nausea or vomiting, for 2.7 months after the treatment of the primary lesion. The Karnofsky performance status (KPS) score was 70 and no pathological reflexes were observed on physical examination. Brain MRI revealed two nodules (0.35 and 0.8 cm in diameter) in the right cerebellar hemisphere. The signal was slightly lower in the unenhanced T1-weighted images and slightly higher in the T2-weighted images. The lesions showed clear ring-like, uniform hardening on signal-enhanced scans, and mild edema was observed surrounding the larger lesion. The diagnosis was of a cerebellar metastasis from the esophageal cancer following treatment (Figs. 3–5). Following whole brain radiotherapy of a total dose of 30 Gy/20 fractions of 1.5 Gy per fraction by hyperfractionation (two fractions per day), the patient’s occipital headache was relieved, however, the neck numbness was aggravated. A cervical MRI examination revealed enlargement of the medulla oblongata and spinal cord, with slightly increased T1 and T2 signals. A signal-enhanced scan showed heterogeneous enhancement in a region considered to be the metastasis. The patient continued with the planned whole brain radiotherapy, together with nedaplatin chemotherapy (40 mg on days one to three of the radiotherapy period). At the end of the first period of chemotherapy, the patient’s occipital headaches were alleviated and the neck numbness was markedly improved. One month later, the patient’s head and neck symptoms had further improved and a neck MRI revealed that the abnormal signal foci in the medulla oblongata and cervical cord had also markedly improved (Figs. 6 and 7). To strengthen the effects of the treatment, the patient was treated with palliative radiotherapy (total dose of 26 Gy/13 fractions of 2 Gy per fraction) to the cervical spinal cord. Following treatment, the patient’s head and neck pain and numbness symptoms had almost disappeared. The patient was discharged with oral etoposide (VP-16) capsules (50 mg daily for 20 days).

### Prognosis of patient and follow-up

Once discharged, the patient refused to return to the hospital for further treatment and follow-up. The occipital headache symptoms recurred, and the patient’s condition gradually deteriorated. The patient succumbed to systemic organ failure on April 16, 2010, six months after the diagnosis of brain metastasis.

## Discussion

A limitation of this case was the lack of autopsy tissue pathology and cerebrospinal fluid cytology to support the diagnosis. A review of the literature on the brain metastasis of esophageal cancer found that, with the exception of cases that underwent surgical resection of the brain metastatic lesions, the patients were treated with chemotherapy and diagnosed by imaging (4–6,8–12). Evidence from MRI and improvements in the clinical symptoms of the patient following radiotherapy determine the diagnosis of cerebellar, brainstem or spinal cord metastasis of esophageal cancer. The most common clinical signs and chief complaints for brain metastases of esophageal carcinoma are weakness (58%), headache (28%), epilepsy (22%) and cerebellar dysfunction (14%) (8).

In the present case, following the treatment of the primary esophageal tumor, the main positive central nervous system signs were fatigue, headaches and neck pain. Takeshima *et al* (11) reported on the characteristic cyst structure of metastatic brain tumors of squamous cell esophageal carcinoma. On T1-weighted images, following the administration of the gadolinium with diethylenetriaminepentaacetic acid contrast agent, the majority of lesions appear as thin-walled cysts with slightly higher signals than those of the brain tissues. Signals from the interior of the cysts are similar to those of the cerebrospinal fluid, however, the walls of the cystic tumors exhibit high signal reflection (11). In the present case, the brain MRI did not exhibit thin-walled cysts, but showed the appearance of solid nodules. Esophageal cancers may become necrotic, characterized by rapid growth, solid tumors that have an inadequate blood supply and the development of focal necrosis in the center of the tumor, resulting in a cyst-like appearance. Prior to the end of the 20th century, brain metastases of esophageal carcinoma were confirmed primarily by pathological examination at autopsy. Central nervous system metastasis of esophageal cancer is also extremely rare. Ogawa *et al* reported brain metastasis in 36 (1.4%) of 2,554 esophageal cancer patients (8). Gabrielsen *et al* (4) observed that the incidence of brain metastasis of esophageal cancer was 3.6% (4), and Weinberg *et al* (5) reported 27 cases of brain metastasis (1.7%) in 1,588 esophageal cancer patients. There are no literature reports of brainstem and spinal cord metastasis of esophageal cancer.

Due to the low incidence of brain metastasis, there is generally no requirement for an MRI examination of the brain and cervical spine of patients with esophageal cancer; Japanese studies have even opposed the routine brain CT examination of patients with esophageal cancer (4). Ogawa *et al* (8) suggested that with the rapid development of medical imaging technology, particularly the application of MR and the use of enhanced contrast agents in neuroimaging, faster and earlier detection of brain metastasis of esophageal carcinoma has become possible (5,8,9). Currently, for the detection and evaluation of tumor lesions, MRI is superior to CT. Therefore, the prompt use of MRI is necessary for the early detection of brainstem and spinal cord metastatic lesions. Certain studies have considered the use of adjuvant chemotherapy following the resection of esophageal carcinoma to increase the risk of brain metastasis (6,12).

It has been confirmed that the distant metastasis of esophageal cancer depends predominantly on the lymphatic and blood systems. The most common pathway for brain metastasis of esophageal cancer is hematogenous dissemination through the arterial circulation. In 1940, the Batson venous plexus was proposed as a pathway for the brain metastasis of esophageal carcinoma (13). However, the role of the vertebral venous system in the brain metastasis of esophageal cancers is supported by a more recent study (8). The present case may be more illustrative of the importance of the vertebral vascular system for cerebellar, brainstem and spinal cord metastases from esophageal cancer, as all three regions are anatomically associated with the same vertebral vascular system, which is distinct from the venous system of the thoracic and lumbar spine and brain.

Brain metastases are often treated with multiple therapies, including surgery, radiation and chemotherapy. Complete removal of the lesions is the goal of surgical treatment and may improve survival rates. However, this generally only applies to patients with a high KPS score or to those with single, solitary tumors (8,10).

Ogawa *et al* (8) reported that patients treated with surgery and radiation treatment have a median survival time of 9.6 months, while patients receiving only radiation treatment have a median survival time of only 1.8 months. In the present case, due to multiple metastases to the cerebellum, brainstem and spinal cord, a combined treatment of local irradiation and chemotherapy was used. The symptoms were significantly improved following treatment, with a survival time of 3.9 months.

Brain metastasis of esophageal cancer may also involve the brainstem and spinal cord, for which local and comprehensive treatments may improve the efficacy of therapy and therefore prolong survival time.
